# Effects of sodium selenite and selenium-enriched yeast on cardiometabolic indices of patients with atherosclerosis: A double-blind randomized clinical trial study

**DOI:** 10.34172/jcvtr.2021.51

**Published:** 2021-12-05

**Authors:** Mahdiyeh Khabbaz Koche Ghazi, Samad Ghaffari, Mohammad Naemi, Rezvaniyeh Salehi, Mohammadreza Taban Sadeghi, Meisam Barati, Alireza Namazi Shabestari, Behnam Kafil, Naimeh Mesri Alamdari, Hamid Soleimanzadeh, Ali Tarighat-Esfanjani, Neda Roshanravan

**Affiliations:** ^1^Nutrition Research Center, Faculty of Nutrition and Food Sciences, Tabriz University of Medical Sciences, Tabriz, Iran; ^2^Cardiovascular Research Center, Tabriz University of Medical Sciences, Tabriz, Iran; ^3^Student Research Committee, Department of Cellular and Molecular Nutrition, Faculty of Nutrition and Food Technology, Shahid Beheshti University of Medical Sciences, Tehran, Iran; ^4^Department of Geriatric Medicine, School of Medicine, Tehran University of Medical Sciences, Tehran, Iran; ^5^Endocrine Research Center, Tabriz University of Medical Sciences, Tabriz, Iran; ^6^Student Research Committee, Department of Nutrition, School of Public Health, Iran University of Medical Sciences, Tehran, Iran; ^7^Department of Applied Chemistry, Faculty of Chemistry, University of Tabriz, Tabriz, Iran

**Keywords:** Atherosclerosis, Glycemic Indices, Lipid Profile, Selenium

## Abstract

***Introduction:*** Atherosclerosis and related cardiovascular diseases (CVDs) are the major causes of mortality worldwide. The available reports regarding the effects of selenium (Se) supplementation in the realm of atherosclerosis have been equivocal. The present investigation is aimed to assess the effects of sodium selenite and Se-enriched yeast supplementation on metabolic parameters among atherosclerotic patients.

***Methods:*** In this double-blind placebo-controlled randomized clinical trial, 60 patients diagnosed with atherosclerosis were randomly allocated into either 200 μg/day selenite, yeast, or placebo groups for eight consecutive weeks. Serum levels of lipid profile and glycemic indices were measured at the baseline and end of the intervention.

***Results:*** There were no significant within-or between-group changes in levels of total cholesterol, triglycerides, high-density lipoprotein cholesterol (HDL-c), fasting blood sugar, insulin, and homeostatic model assessment for IR throughout the study (*P* ≥0.05). Only the low density lipoprotein cholesterol (LDL-c) levels were significantly lower in the yeast group in comparison with the placebo group (*P* = 0.015).

***Conclusion:*** The administration of Se-enriched yeast is significantly effective in decreasing LDL-c levels in patients with atherosclerosis. Additional clinical trial studies investigating the effect of Se administration on glucose homeostasis parameters and lipid profiles in atherosclerotic patients are suggested for a more definitive conclusion.

## Introduction

Selenium (Se), as an essential trace mineral, has a critical role in human health. The physiologic function of Se is dependent on the seleno-proteins, in which Se is present as seleno-cysteine amino acid.^1^ Se function is required for antioxidant defense, prevention of inflammation, thyroid hormone generation, DNA synthesis, reproduction, and cancer cell death.^2,3^ The most crucial function of Se is catalyzing the conversion of H_2_O_2_, lipid, and phospholipid hydroperoxides into safe products, mainly through Se-dependent glutathione peroxidase (GPXs), which is related to the antioxidant effects of Se.^4^ This process prevents oxidative damage to the lipids, lipoproteins, and DNA. It also maintains the membrane integrity, which is finally associated with cardiovascular diseases (CVDs) such as atherosclerosis.^5,6^ Oxidative stress is recognized as the crucial factor leading to atherosclerosis and related CVDs. Atherosclerosis, as a multifactorial and progressive disorder of the arterial wall, is the major cause of mortality and morbidity worldwide.^7^ It results from the deposition of fatty materials and cholesterol in the arterial intima, mainly caused by oxidative modifications of low-density lipoprotein (LDL). The modified lipoprotein causes an increase in the expression of cell adhesion molecules on the endothelial cells, which results in the recruitment of leukocyte into the sub-endothelial space. These lipid-laden macrophages are called foam cells whose presence in the arterial wall is a hallmark of early atherosclerotic lesion.^8,9^

Over the last three decades, considerable attention has been given to Se, because it may prevent atherosclerosis and CVDs by inhibiting oxidative modification of lipids, inflammation, endothelial dysfunction, vascular cell apoptosis, and vascular calcification.^10-13^

Since the inorganic Se has low bioavailability in organisms, finding a proper source of Se as a dietary supplement is pivotal.^14^ The most bioavailable form of Se is organic Se complexes and Se-containing amino acids.^15^ One of these dietary agents is Se-enriched yeast, which possesses higher amounts of intracellular organic Se, in which Se replaces sulfur in protein.^16^ In a Se-enriched media, yeast absorbs large amounts of Se and incorporates it into organic Se- containing components. Numerous human and animal studies has investigated the effect of Se on cardiovascular endpoints in different metabolic diseases.^17-20^

Recent findings on the association of the Se supplementation with cardio-metabolic parameters have been non-conclusive. However, in a clinical trial conducted on French adults, supplementation with a combination of antioxidants including 120 mg vitamin C, 30 mg vitamin E (all-*rac*-a-tocopherol), 6 mg b-carotene, 100 μg selenium (as selenium-enriched yeast), and 20 mg zinc (as gluconate) during 7.5 years, had no effect on total cholesterol level in participants. Furthermore, the prevalence of hypercholesterolemia and hypertriglyceridemia was higher in women who received supplements compared with those who received placebo.^21^ Another clinical trial with high-Se yeast 100 and 200 μg/day for six months indicated a modestly beneficial effect on the lipid profile of the subjects with low serum levels of Se. Se on glycemic indices was also investigated in different metabolic disorders, and the results are contradictory. ^22-25^

AS mentioned above, the precise effect of selenium on the cardio metabolic parameters has not yet been detected, and the results of the investigations are inconsistent. On the other hand, based on the available data, there is no study investigating the effect of selenium-enriched Yeast, which is the active form of the selenite, in patients with atherosclerosis. So this randomized controlled trial (RCT) aims to investigate, for the first time, effects of Se-enriched yeast on cardiovascular indices, and to compare it with sodium selenite in patients with atherosclerosis.

## Materials and methods

### Study design and subjects

This study was a double-blind placebo-controlled RCT that was conducted on patients with atherosclerosis. The subjects involved in the study were chosen from Shahid Madani heart Hospital of Tabriz University of Medical Sciences, Tabriz, Iran, between July and October 2019. Two cardiologists, through careful examinations and angiography, confirmed atherosclerosis.

The inclusion criteria were adults with atherosclerosis, newly diagnosed, through angiography with more than 50% stenosis at least in one artery, aged between 18 and 80 years, with body mass index (BMI) between 25–35 kg/ m^2^.

The exclusion criteria include: Having chronic liver or kidney disorders, infectious diseases, pregnancy or breastfeeding, consuming any drug which may influence lipid and glucose metabolism, dietary supplements, and antioxidants over six months before the study. Furthermore, if less than 90% of placebo or supplements was consumed, the data from the participants were not considered in the analysis.

At the baseline of the study, the written informed consent was received from all participants.

This study was approved by the Ethics Committee of Tabriz University of Medical Sciences (Ethical code: IR.TBZMED.REC.1398.1187). This clinical trial study is registered in the Iranian Registry of Clinical Trials (IRCT ID: IRCT20110123005670N28) and is accessible on the IRCT website. Furthermore, this manuscript was reported according to CONSORT 2010 guidelines for reporting clinical trials.

The sample size of the study was defined based on the mean ( ± standard deviation [SD]) of GPX of Ying Hu, et al, study ^24^ with a confidence interval of 95% and power of 80 %, and estimation 10% of dropouts. The total sample size for each group (yeast, selenite, and placebo groups) was 20 patients. Using a randomized block procedure, Patients were randomly allocated to one of the three treatment groups, including the yeast group (n = 20), selenite group (n = 20), and placebo group (n = 20). The investigators and participants were blinded to allocations. The supplements, including Se-enriched yeast capsules and sodium selenite, were synthesized at Nutrition Research Center, Tabriz University of Medical Sciences, Iran. Se-enriched yeast is produced by growing *Saccharomyces cerevisiae*in Se-rich media. Its preparation and optimization of culture conditions are reported in detail earlier in the studies.^15,16^

Based on a similar study ^24^ and tolerable upper levels of Se, the Se-enriched yeast and sodium selenite intervention doses were determined to be about 200 µg/ day. Subjects of the yeast group took one capsule of 200 µg/day Se-enriched yeast every day (30 min before dinner), the selenite group took a 200 µg/day capsule of sodium selenite every day (30 min before dinner) and also the placebo group took the 200 µg/day starch capsules which contain cellulose, silicon dioxide, and starch (30 min before dinner) for eight consecutive weeks. All intervention capsules and placebo were given to the groups every week. The shape, size, and color of capsules in all study groups -were alike. The amounts of capsules returned by subjects were taken as the criteria of adherence to interventions. The consumption of ≥90% of the supplements was defined as good adherence.

At the baseline and the end of the intervention, five-milliliter of fasting blood samples were collected from all of the patients. They were centrifuged at 3000 rpm for 10 min and then the serum samples were stored at -80^°C^ to further analysis.

Serum total cholesterol (TC), triglycerides (TG), low-density lipoprotein cholesterol (LDL-c), high-density lipoprotein cholesterol (HDL-c), and fasting blood sugar (FBS) were determined using the enzymatic colorimetric method and commercial kits (Pars Azmoon Co., Tehran, Iran). Insulin concentration was measured, by an enzyme-linked immunosorbent assay (ELISA) kit (Monobind, USA) based on the manufacturer’s instructions. The homeostatic model assessment for IR (HOMA-IR) was defined by the following formula: HOMA-IR = [fasting insulin (μIU/mL) × fasting glucose (mg/dL)]/405.

A digital automatic blood pressure monitor (Omron, Japan) was used to measure blood pressure after 10 minutes of resting period. The weight and height of subjects were measured with minimum clothes and no shoes, using a calibrated scale 1and stadiometer (Seca, Hamburg, Germany) to the nearest 0.1 kg and 0.1 cm, respectively. BMI was computed as weight (kg)/height (m^2^).

### Statistical analysis

The Kolmogorov Smirnov test was used to check the normality of the distribution of numeric variables. The result of the test showed non-normal distribution of data (*P* < 0.05). Numerical data were presented as mean (SD) and categorical variables reported as frequency (percentage).

For assessing the differences among groups at baseline and end of the study, the Kruskal-Wallis test and Chi-Square test were used for categorical and numerical variables. Wilcoxon Signed Ranks Test was used for assessing within-group changes for non-normal distribution of data. The statistical software SPSS version 23 (SPSS Inc. IL, Chicago, USA) was used for data entry and analysis. P-value < 0.05 was considered statistically significant.

**Result**s

**Figure 1:**
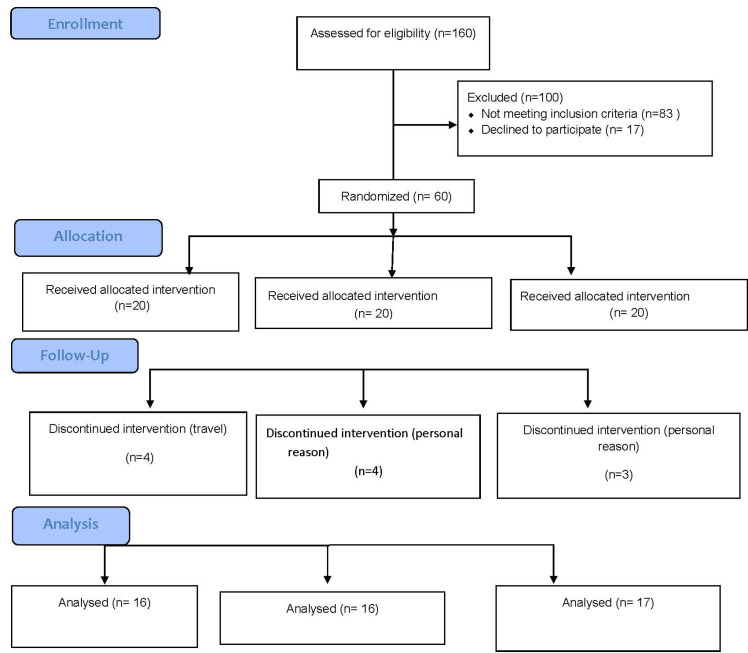
Study flow diagram

The flowchart of the study and design is given in Figure 1. The study was carried out with a total of 49 patients. Three patients in the placebo group dropped out because of surgery, four patients from the yeast group stopped taking part in the study for personal reasons, and four patients from the selenite group were pulled out due to travel. According to participants’ reports, supplementation with Se-enriched yeast and sodium selenite did not have any side effects or symptoms. As presented in Table 1, at baseline of the study there were no significant differences in general and demographic characteristics among studied groups. Furthermore, the patients in all groups are not different in health status, tobacco consumption and the other demographic characteristics (data are not shown here).

**Table 1:** Baseline characteristics of the study participant

**Variables**		**Groups**			***P*** **value** ^a^
		**Selenium-enriched Yeast** **(n=16)**	**Sodium Selenite** **(n=16)**	**Placebo** **(n=17)**	
Age (Year)		59.06 ± 8.55	58.62 ± 9.68	53.58 ± 13.75	0.287
Weight(kg)		81.03 ± 11.35	81.18 ± 9.43	83.71 ± 13.73	0.759
Height(m)		1.68 ± 0.07	1.70 ± 0.05	1.70 ± 0.08	0.868
BMI(kg/m^2^)		28.56 ± 3.63	27.86 ± 2.21	28.29 ± 3.07	0.774
SBP(mm Hg)		110.62 ± 15.37	111.87 ± 15.90	119.70 ± 13.04	0.096
DBP (mm Hg)		70.90 ± 8.98	76.25 ± 7.41	75.29 ± 10.22	0.251
Gender	Male	12 (75)	16 (100)	15 (88.2)	0.097^b^
	Female	4 (25)	0 (0)	2 (11.8)	

Abbreviation: BMI, body mass index; SBP, systolic blood pressure; DBP, diastolic blood pressure. Qualitative variables are reported as frequency (percent). Quantitative variables are reported as mean ± SD. ^a^ *P* value is reported based on Kruskal-wallis test; ^b^ *P* value is reported based on Chi-Square test. *P < 0.05 considered as statistically significant

Changes in biochemical parameters of patients are presented in Table 2. There were no significant within-or among-group changes in blood pressure, lipid, and glucose profile throughout the study (*P*≥0.05). Only the LDL-C levels were significantly different among groups. the LDL-C level was lower in the yeast group in comparison with the placebo group (*P* = 0.015). The after intervention LDL-c levels were 61.87 ± 16.89 mg/dL, 77.37 ± 36.28 mg/dL and 82.88 ± 21.79 mg/dL in Selenium-enriched, sodium Selenite and placebo group, respectively.

**Table 2:** Comparison of biochemical variables between study groups^a^

**Variables**		**Groups**			***P*** **value** ^c^
		**Selenium-enriched** **Yeast** **(n=16)**	**sodium** **Selenite** **(n=16)**	**Placebo** **(n=17)**	
SBP(mm Hg)	Before	110.62 ± 15.37	111.87 ± 15.90	119.70 ± 13.04	0.096
	After	119.25 ± 17.59	119.64 ± 20.04	124.41 ± 19.27	0.775
	*P*value^b^	0.400	0.095	0.273	
	Change(mm Hg)	8.62 ± 2.33	7.50 ± 4.37	4.70 ± 3.79	0.714
DBP(mm Hg)	Before	70.90 ± 8.98	76.25 ± 7.41	75.29 ± 10.22	0.251
	After	74.93 ± 7.53	74.07 ± 10.58	77.17 ± 7.40	0.512
	*P*value^b^	0.101	0.572	0.452	
	Change(mm Hg)	4 ± 2.44	-2.35 ± 2.89	1.88 ± 2.45	0.239
FBS(mg/dL)	Before	115.43 ± 37.09	103.87 ± 22.51	115.17 ± 45.64	0.855
	After	116.37 ± 43.01	96.37 ± 11.05	115.05 ± 45.08	0.523
	*P* value^b^	0.780	0.173	0.831	
	Change(mg/dL)	0.93 ± 5.92	-7.50 ± 5.57	-0.11 ± 8.70	0.654
Insulin (μIU/mL)	Before	13.68 ± 6.55	16.81 ± 11.22	12.31 ± 5.18	0.875
	After	13.96 ± 5.9	10.41 ± 3.63	13.21 ± 6.74	0.192
	*P*value^b^	0.999	0.118	0.758	
	Change (μIU/mL)	0.15 ± 0.05	-11.03 ± 5.31	-1.43 ± 0.04	0.283
HOMA_IR	Before	4.09 ± 0.66	5.41 ± 2.52	3.53 ± 0.56	0.768
	After	3.59 ± 0.83	2.66 ± 0.41	3.54 ± 0.83	0.884
	*P*value^b^	0.401	0.110	0.388	
	Change	0.36 ± 0.02	-4.72 ± 0.18	-0.38 ± 0.04	0.271
TC(mg/dL)	Before	123.56 ± 13.96	135.56 ± 33.38	144.23 ± 36.46	0.067
	After	125.06 ± 24.46	142.75 ± 43.26	148.76 ± 30.69	0.094
	*P*value^b^	0.678	0.587	0.289	
	change(mg/dL)	1.50 ± 0.82	7.18 ± 0.46	4.52 ± 0.54	0.879
TG(mg/dL)	Before	153.5 ± 55.34	166.18 ± 90.13	196.58 ± 31.37	0.790
	After	172.56 ± 14.99	153.31 ± 66.15	206.64 ± 75.58	0.933
	*P*value^b^	0.755	0.535	0.831	
	change(mg/dL)	19.06 ± 0.71	-12.87 ± 4.78	10.05 ± 3.63	0.627
LDL(mg/dL)	Before	60.56 ± 13.89	70.12 ± 28.98	72.11 ± 25.59	0.342
	After	61.87 ± 16.89	77.37 ± 36.28	82.88 ± 21.79	0.015
	*P*value^b^	0.979	0.234	0.078	
	change(mg/dL)	1.31 ± 0.71	7.25 ± 1.24	10.76 ± 3.25	0.472
HDL(mg/dL)	Before	32.56 ± 6.2	35 ± 12.63	37 ± 8.299	0.175
	After	32.5 ± 7.66	34.62 ± 9.44	34.94 ± 7.77	0.682
	*P*value^b^	0.900	0.917	0.331	
	change(mg/dL)	-0.06 ± 0.08	-0.37 ± 0.04	-2.05 ± 0.02	0.518

Abbreviation: SBP, systolic blood pressure; DBP, diastolic blood pressure; FBS, fasting blood sugar; HOMA-IR, Homeostatic Model Assessment of Insulin Resistance; TC, total cholesterol; TG, triglyceride; LDL, low density cholesterol; HDL, high density cholesterol. ^a^Data are presented as Mean ± SD. ^b^ *P* value is reported based on Wilcoxon signed Ranks Test. ^c^ *P* value is reported based on Kruskal-wallis Test. *P < 0.05 considered as statistically significant.

## Discussion

This clinical trial studied the effects of sodium selenite and Se-enriched yeast supplementation on metabolic indices of patients with atherosclerosis. According to our results, Se-enriched yeast and Se did not change the glycemic indices and lipid profile of patients with atherosclerosis compared to placebo, except for LDL-c. The LDL-c serum levels were significantly lower in the yeast group in comparison with the others. Overall, the findings of the present study indicated that Se supplementation in sodium selenite and Se-enriched yeast form seemed to have no beneficial effects on plasma lipids and glycemic indices in atherosclerotic patients. Although it modified the LDL-c levels in comparison with the placebo group, the results of the present study were in line with that of a recent meta-analysis in which 11 RCT papers showed that the supplementation with Se had no beneficial effect on TC and HDL-c, and a marginal lowering effect on TG level.^19^ In another meta-analysis of five RCT, which assessed the effect of 200 µg/day Se supplementation on glucose metabolism and lipid profile among patients with diseases related to metabolic syndrome (MetS), Se did not affect fasting plasma glucose (FPG), HOMA-IR, and lipid profiles.^20^ However, in an animal study it was found thatsupplementation with 1 ppm Se in rats for 3 months increased the LDL-R activity as well as mRNA expression of it significantly. Furthermore, Total cholesterol and LDL levels decreased significantly. This study demonstrated that Se supplementation upregulated the LDL-R activity as well as mRNA expression. These findings emphasize the therapeutic potential of Se supplementation in lipid metabolism and its relationship pathways.^21^

LDL-c is the most important carrier of cholesterol in the circulation and is closely associated with atherosclerotic plaque formation. Low-density lipoprotein receptor intercedes the elimination of LDL from blood through binding to apolipoprotein B (apo B) which contains the ligand-binding domain for binding of LDL to LDL-R site, which enables the removal of LDL from circulation. ^22^

It seems that upregulation of LDL receptor expression during selenium supplementation might have offered more receptor sites for LDL binding and its clearance from the circulation. So, in the present study Se-enriched yeast, which is the active form of Se, might decrease the LDL-c levels for the mentioned reason.

It seems that the Se status of the participants, the dosage of Se, and study duration are important factors that determine the Se association with markers of glucose and lipid metabolism.

In a recent meta-analysis of 12 RCTs, Se supplementation did not improve the glycemic indices, such as FPG, insulin, and HOMA-IR in patients with metabolic disease. The biological impact of Se supplementation on glycemic indices can be attributed to the antioxidant properties of Se. Seleno-proteins with antioxidant properties contain GPX, which decreases hydrogen peroxide, lipid, glucose, and phospholipid hydroperoxides.^6,18^ Se may improve insulin levels by inhibiting the expression of cyclooxygenase-2 and P-selectin and preventing the production of inflammatory cytokines such as tumor necrosis factor-α and interleukin-1.^1,17^ Although the underlying mechanism of Se effects on plasma lipids are not clear, it seems that in several pathways Se or seleno-proteins interact with both lipids and lipoproteins. Se is incorporated into seleno-proteins as seleno-cysteine (Sec) which is involved in the modulation of Apo lipoprotein E production, cholesterol biosynthesis, and metabolism.^2,26^

## Conclusion

In summary, Se supplementation in both forms seemed to have no beneficial effects on plasma lipid levels, except for LDL-c and glycemic indices of atherosclerotic patients. Although this study, which was conducted on patients with atherosclerosis, was the first study that compares the effects of Se in both sodium selenite and Se-enriched yeast form, it had some limitations. We did not assess the effect of Se onothercardio metabolic risks. Furthermore, the baseline Se status of the population and also the after-intervention amounts of selenium in serum was not measured, and the duration of intervention was limited. Further clinical trials are needed in larger populations to quantify these effects.

## Acknowledgments

The authors would like to thank the vice chancellor of research, Tabriz University of Medical Sciences. The authors also are grateful to all patients who participated in this research project.

## Competing interests

Authors have no conflict of interest.

## Ethics approval

The written consent was obtained from participants of study. This study was approved by the Ethics Committee of Tabriz University of Medical Sciences (Ethical code: IR.TBZMED.REC.1398.1187). This clinical trial study is registered in the Iranian Registry of Clinical Trials (IRCT ID: IRCT20110123005670N28) and is accessible on the IRCT website

## Funding

This research project was funded by the Research Vice-Chancellor, Tabriz University of Medical Sciences under the grant (62912).
